# Droplet digital PCR vs. quantitative real time-PCR for diagnosis of pulmonary and extrapulmonary tuberculosis: systematic review and meta-analysis

**DOI:** 10.3389/fmed.2023.1248842

**Published:** 2023-08-07

**Authors:** Edinson Dante Meregildo-Rodriguez, Martha Genara Asmat-Rubio, Gustavo Adolfo Vásquez-Tirado

**Affiliations:** ^1^Escuela de Medicina, Universidad César Vallejo, Trujillo, Peru; ^2^Escuela de Posgrado, Universidad Privada Antenor Orrego, Trujillo, Peru; ^3^Escuela de Medicina, Universidad Privada Antenor Orrego, Trujillo, Peru

**Keywords:** tuberculosis, polymerase chain reaction, diagnosis, systematic review, meta-analysis

## Abstract

Tuberculosis is a rising global public health emergency. Then, it is a priority to undertake innovations in preventive, diagnostic, and therapeutic methods. Improved diagnostic methods for tuberculosis are urgently needed to address this global epidemic. These methods should be rapid, accurate, affordable, and able to detect drug-resistant tuberculosis. The benefits of these new diagnostic technics include earlier diagnosis and treatment, improved patient outcomes, and reduced economic burden. Therefore, we aimed to systematically review the diagnostic performance of droplet digital PCR (ddPCR)—a third-generation PCR—compared with quantitative Real Time-PCR (qPCR) for diagnosing pulmonary and extrapulmonary tuberculosis. We included 14 diagnostic accuracy test studies performed in Asia, Europe, and Latin America, 1,672 participants or biological samples, and 975 events (pulmonary or extrapulmonary tuberculosis). Most of the included studies had a low risk of bias (QUADAS-C tool). Sensitivity and specificity were lower for ddPCR [0.56 (95% CI 0.53–0.58) and 0.97 (95% CI 0.96–0.98), respectively] than for qPCR [0.66 (95% CI 0.60–0.71) and 0.98 (95% CI 0.97–0.99), respectively]. However, the area under the ROC curve (AUC) was higher for ddPCR than for qPCR (0.97 and 0.94, respectively). Comparing both AUCs using the Hanley & McNeil method, we found statistically significant differences (AUC difference of 4.40%, *p* = 0.0020). In the heterogeneity analysis, we found significant differences between both techniques according to the continent of origin of the study and the location of tuberculosis (pulmonary or extrapulmonary disease). The AUCs of both methods were similar in pulmonary tuberculosis. However, for extrapulmonary tuberculosis, the AUC was higher for ddPCR. We found some limitations: (1) significant heterogeneity of the studies, and (2) we could not perform subgroup analyses according to other relevant variables, such as the age and sex of the participants. Nonetheless, this study is the first meta-analysis that shows that ddPCR has a comparable diagnostic performance than qPCR for pulmonary tuberculosis. However, for extrapulmonary tuberculosis, ddPCR has a better discriminant capacity to differentiate between patients with and without extrapulmonary tuberculosis. We conclude that ddPCR is likely the best diagnostic technic for tuberculosis diagnosis, especially for extrapulmonary tuberculosis. More studies are still needed yet.

**Systematic review registration:**

https://www.crd.york.ac.uk/prospero/display_record.php?ID=CRD42022382768, CRD42022382768.

## 1. Introduction

Tuberculosis is currently the leading global cause of death due to infectious diseases among adults. It has been considered a rising global public health emergency in recent decades (1, 2). Tuberculosis is classified as a pulmonary (PTB) or extrapulmonary disease (EPTB). The former is the most common. EPTB refers to disease involving organs other than the lungs (e.g., pleura, lymph nodes, abdomen, genitourinary tract, skin, joints and bones, or meninges). A patient with both pulmonary and EPTB is classified as a case of PTB. Miliary TB is classified as PTB because there are lesions in the lungs. However, the miliary disease has also been classified as both extrapulmonary and pulmonary TB (3). Of the 7,174 cases reported in United States in 2020, EPTB (without proven pulmonary involvement) accounted for more than 20%. Tuberculous involving both pulmonary and extrapulmonary sites was reported in approximately 80% of cases (4). Worldwide, incidence estimates are hampered by underreporting and imprecise diagnostic criteria. EPTB accounts for 15% of the 7.3 million incident cases reported in 2018 (5).

The strategies implemented in public health against tuberculosis have saved millions of lives. However, little has been achieved to control, and less to eradicate, tuberculosis (1). Drug-resistant tuberculosis is becoming one of the diseases caused by the world’s deadliest pathogens, responsible for a quarter of deaths due to antimicrobial resistance (5–7). Then, controlling tuberculosis worldwide remains critical (8). It is, therefore, a priority to undertake new, ambitious, and radical actions to confront this potentially curable pathogen, which continues to be one of the biggest global public health problems (1). These innovative actions could occur in preventive, diagnostic, and therapeutic methods. In this sense, it is very reasonable to focus on the exhaustive evaluation of the new diagnostic tools. EPTB has extremely low acid-fast bacilli (AFB) concentrations that traditional diagnostic methods cannot detect (9, 10).

Molecular techniques overcome the limitations, insensitivity, and time loss of AFB staining and culture for diagnosing paucibacillary EPTB (8). Innovative diagnostic tools with increased specificity, sensitivity, and automation have been developed recently. For example, molecular detection of genes significantly reduces the time required for drug susceptibility testing from months to hours to complete phenotypic drug susceptibility assays (1, 8). Digital droplet PCR (ddPCR)—a third-generation PCR-based technology—is one of the most recently developed diagnostic techniques.

Quantitative Real-Time PCR (qPCR) and ddPCR share similarities but differ in some aspects. (1) *Principles.* qPCR detects and amplifies target DNA or RNA in real-time using fluorescent probes or DNA-binding dyes to monitor the amplification signal directly proportional to the target nucleic acid present. ddPCR partitions a sample into thousands of droplets containing a few copies of the target genetic material. The amplification occurs in each droplet, and the endpoint signal is measured after the amplification completion. The absolute quantification of target molecules is achieved by counting the positive and negative droplets. (2) *Data Analysis.* qPCR data is analyzed using threshold cycle (Ct) values, representing the amplification cycle at which the fluorescence signal reaches a specific threshold. The Ct values calculate the amount of target nucleic acid using standard curves or comparative methods. ddPCR data analysis determines the fraction of positive droplets and converts it into a concentration of target molecules using Poisson statistics and quantifies target molecules without needing external standards or calibration curves. (3) *Throughput.* qPCR can process more samples simultaneously in a single run using 96 or 384 well plates, and it is suitable for high-throughput screening. ddPCR typically has a lower throughput, as each sample needs to be partitioned into individual droplets, limiting the number of samples processed in a single run (11–14).

The information available on this innovative tool (ddPCR) seems to be promising. However, to date, no study has systematically reviewed the evidence on the diagnostic performance of this method. Given the current limitation of standard methods for diagnosing TB, our study aims to fill this research gap. Therefore, we aim to synthesize the available evidence on the diagnostic performance of ddPCR compared to quantitative Real Time-PCR (qPCR) in diagnosing EPTB. This research contributes to the field by assessing systematically and comprehensively the diagnostic accuracy of ddPCR for TB, comparing the diagnostic accuracy of ddPCR to qPCR, and investigating the factors that may affect its accuracy. Our results have important implications for developing and implementing ddPCR as a diagnostic tool for TB. If ddPCR is more accurate than qPCR, it could significantly impact the early diagnosis and treatment of TB, especially in low-resource settings.

## 2. Materials and methods

We carried out this systematic review following the recommendations of the Cochrane Handbook for Systematic Reviews (15), PRISMA-DTA (16), and AMSTAR 2 (17) guidelines. We registered the protocol in PROSPERO (CRD42022382768) and searched in MEDLINE (PUBMED), Scopus, EMBASE, Web of Science, ScienceDirect, Google Scholar, and Cochrane Library.

We followed a PICO strategy [population: patients with “tuberculosis”; intervention: “droplet digital PCR” OR “digital PCR”; comparator: “quantitative PCR” OR “conventional PCR” OR “RT-PCR” OR “real time-PCR,” OR quantitative Real Time-PCR; outcome: “diagnostic accuracy” OR “sensitivity” OR “specificity” OR “positive predictive value” OR “negative predictive value” OR “area under the ROC curve”] and combined keywords, free terms, and controlled vocabulary terms (e.g., MeSH and Emtree) using Boolean operators. As a means to collect as many studies as possible, we used a sensible search strategy, mainly combining the terms “tuberculosis” AND “digital” AND “Polymerase Chain Reaction” (Supplementary materials).

We included studies published up to March 31, 2023. We screened references from retrieved documents and narrative reviews for additional articles. We excluded case reports, case series, studies not available in full text, and duplicate publications; in this latter case, the paper that reported the most extended follow-up or the largest cohort was included. Besides, we excluded studies on test standardization, analytical validity, or if the reference test was not clearly defined. We report excluded studies and the reasons for their exclusion (Supplementary materials). We did not limit the searches by date or the language of publication.

**Figure 1:**
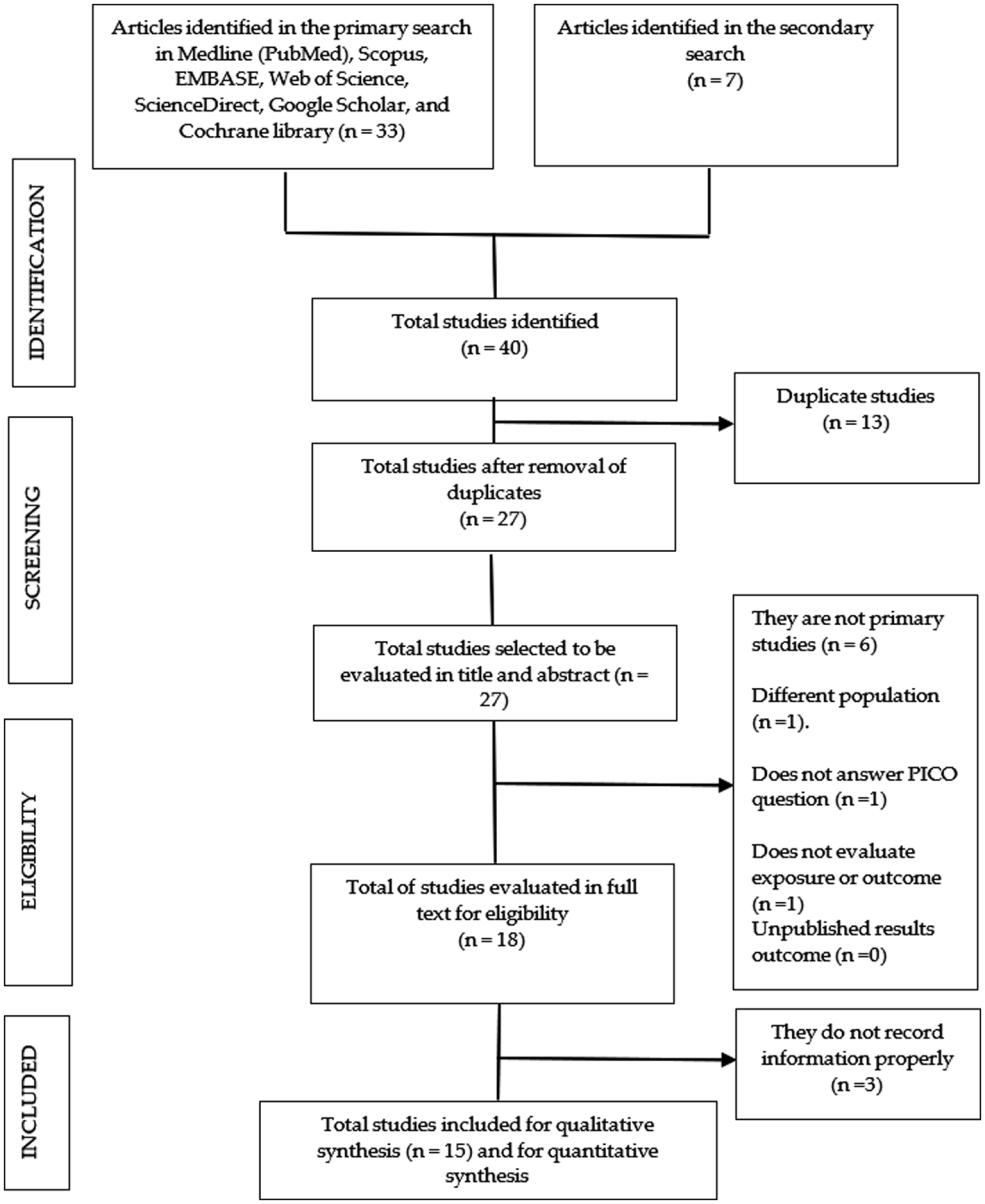
Flow chart of the selection process of the primary studies included.

Two independent and blinded reviewers examined the articles and performed the selection and extraction process. They resolved discrepancies by consensus or by a third researcher in case necessary. The papers found were analyzed using the terms of the PICO strategy and the inclusion and exclusion criteria. The study selection process is detailed in Figure 1.

Relevant information from each paper was extracted and recorded in a spreadsheet: authors’ names, year and country of publication, type of study, number of patients, number of events (tuberculosis), clinical-epidemiological characteristics of the population, the type of biological sample, the reference or standard gold test, and the index test. We considered sensitivity, specificity, positive and negative predictive values, and positive and negative likelihood ratios and area under the ROC Curve (AUC) as diagnostic performance measures.

In the meta-analysis, we combined the number of true positives, false negatives, false positives, and true negatives cases. Studies reporting several different outcomes were considered independent studies. We performed the meta-analysis using RevMan® 5 and MetaDiSc® 1.4 software. We calculated sensitivity, specificity, positive and negative likelihood ratio, diagnostic odds ratio (OR), and area under the ROC curve with their 95% confidence intervals (95% CI). To determine whether ROC curves had statistically significant differences, we compared both AUCs and their standard errors using the Hanley & McNeil method (18).

We assessed heterogeneity using the Der Simonian-Laird Q statistic (χ^2^ distribution). According to our protocol, in case of significant statistical heterogeneity, we would address it according to the recommendations of the Cochrane Manual (15) and choose a random effects model. Likewise, we analyzed the uncertainty (sensitivity analysis) based on the weight percentage of each study on the overall result. Finally, we evaluated the risk of bias using the QUADAS-C instrument. The QUADAS-C tool was developed as an extension of QUADAS-2 to assess the risk of bias in comparative diagnostic test accuracy (DAT) studies (19). The QUADAS-C tool is also useful for systematic reviews of DATs addressing comparative questions. Within systematic reviews of DAT, the QUADAS-C tool can assess risk of bias in test comparisons undertaken in comparative accuracy studies (studies that evaluate two or more index tests) (19, 20).

We performed meta-regressions to compare these diagnostic evaluation parameters according to the continent of origin and clinical form of tuberculosis (pulmonary or extrapulmonary). Forest plot graphs represented the quantitative synthesis. In addition, we report ROC curves with their respective AUCs to distinguish the test with the best-discriminating capacity between healthy and sick patients.

## 3. Results

We found 40 articles, 33 in the primary search and 7 in the secondary search. After removing duplicates, 27 studies remained that were examined for title and abstract. Subsequently, 18 studies remained that were evaluated in full text, 3 of them being excluded because they did not adequately record the information (Supplementary material). Finally, 15 studies remained that were submitted to qualitative synthesis and 14 were included in the meta-analysis (Figure 1).

The 15 included studies pooled 1,852 participants or biological samples and 1,049 events (cases of pulmonary, extrapulmonary, or both forms of tuberculosis). Nine studies were performed in China; South Korea, Japan, Thailand, United Kingdom, Italy, and Peru contributed with one study each.

The types of tuberculosis varied widely, including pulmonary, meningeal, intestinal, and other forms of tuberculosis. The types of analyzed samples also were diverse, including serum or plasma, respiratory secretions (sputum, bronchial or bronchioalveolar lavage, etc.), formalin-fixed and paraffin-embedded (FFPE) samples, cerebrospinal fluid (CSF), pleural fluid, abscess secretions, urine, intestinal biopsies, feces, etc.

Insertion sequences (DNA sequences used for detecting MTB) varied with both methods (qPCR and ddPCR). The most used insertion sequences were IS6110, IS1081, gyrB, rpoB, and Rv3874(CFP10). Furthermore, the reference or gold standard test, varied among the studies. In most studies, the gold standard for diagnosing tuberculosis varied widely. In some studies, it was based on clinical guidelines criteria or a combination of clinical, radiological, microbiological, interferon-γ release assay (IGRA), molecular, histopathological, or even the therapeutic response criteria. However, others defined a case of tuberculosis according to culture or a test based on nucleic acid amplification technologies (NAATs) (Table 1).

**Table 1:** General characteristics of the included studies.

Study, country	Sample	Gold standard	Index test	Sensitivity % (95% CI)	Specificity % (95% CI)
Luo et al. (21), China.	*N* = 102 (sputum). PTB 57 patients.	BACTEC MGIT^™^ culture.	IS6110-ddPCR	95.7 (84.0–99.2)	88.9 (76.7–95.4)
			Xpert-MTB/RIF	91.3 (78.3–97.2)	90.7 (78.9–96.5)
			IS6110-qPCR	84.8 (70.5–93.2)	90.74 (78.9–96.5)
Lyu et al. (22), China.	*N* = 261 (plasma). TB 155 (PTB, EPTB, DTB). No-TB 106.	Combination of several criteria (radiology, microbiology, PCR, AP).	IS6110-ddPCR	40.6 (32.8–42.8)	93.4 (86.9–97.3)
			IS1081-ddPCR	27.1 (20.3–34.8)	93.4 (86.9–97.3)
			IS6110 & IS1081-ddPCR	42.6 (34.7–50.8)	90.6 (83.3–95.4)
Zhao et al. (23), China.	*N* = 605 (respiratory samples). PTB 263.	Combination of several criteria (radiology, ZNS, culture, PCR, IGRA).	IS6110-ddPCR	61.22 (55.00–67.10)	95.03 (92.20–97.10)
Ushio et al. (24), Japan.	*N* = 56 (plasma). TBP 37, sin TB 15, NTM 4.	NAATs in samples positive for ZNS.	IS6110-ddPCR	65.0	93.0
			gyrB-ddPCR	29.0	100.0
Cao et al. (25), China.	*N* = 65 FFPE samples.	Combination of several criteria (clinical diagnosis, radiology, H&E staining, ZN staining, and qPCR).	IS6110-ddPCR	0.75 (0.55–0.89)	0.71 (0.44–0.90)
Li et al. (26), China.	*N* = 101 (CSF). MEC-TB.	Uniform definition of MEC-TB (27).	IS6110-ddPCR	57.4 (44.8–69.3)	97.0 (84.2–99.9)
			gyrB-ddPCR	22.1 (12.9–33.8)	100.0 (89.4–100.0)
Yang et al. (28), China.	*N* = 84 patients (PTB 28, EPTB 28, no-TB 28).	NICE and CCDTGTB guidelines diagnostic criteria.	IS6110-ddPCR vs. IS6110-qPCR	TBP: 28/28 vs. 14/28	28/28 vs. 14/28
				TPE: 28/28 vs. 15/28	28/28 vs. 15/28
				Total: 56/56 vs. 29/56	56/56 vs. 27/56
Li et al. (29), China.	*N* = 310. Patients. Pleural TB 183, no-TB 127. Gender M/F: 149/34.	Diagnostic Criteria for Tuberculosis (WS 288—2017), 2018 (30).	IS6110-ddPCR	57.4 (49.9–64.6)	100.0 (97.1–100.0)
			IS1081-ddPCR	40.4 (33.3–47.9)	100.0 (97.1–100.0)
			IS6110 & IS1081-ddPCR	38.8 (31.7–46.3)	100.0 (97.1–100.0)
			IS6110 OR IS1081-ddPCR	59.0 (51.5–66.2)	100.0 (97.1–100.0)
Antonello et al. (31), Italy.	*N* = 89 biopsies (fresh and FFPE samples). Culture: positive 68, negative 21.	LJ and liquid (MGIT) culture media.	IS6110-ddPCR	98.5 (95.6–100.0)	100.0 (86.3–100.0)
			rpoB-ddPCR	66.2 (54.9–77.4)	100.0 (82.8–100.0)
Rodriguez and Villegas Chiroque (32) Peru.	*N* = 66 patients (no sputum biological samples). TB 23.	Combination of several criteria (clinical diagnosis, radiology, ZNS, culture and therapeutic response).	IS6110-ddPCR	82.6% (62.9–93.0)	95.3 (84.5–98.7)
			IS6110-qPCR	60.9 (40.8–70.8)	93.0 (81.4–97.6)
Nyaruaba et al. (33), China.	*N* = 30 (sputum) samples. TB: 27 patients.	Media 7H9 culture.	IS6110 & IS1081-ddPCR	100	100
			IS6110-qPCR	100	100
Cho et al. (34), Korea.	*N* = 190 respiratory samples. Positive culture (MGIT) 23. PTB 44 patients.	Combination of several criteria (clinical diagnosis, radiology, microbiology, NAATs, and immunology).	Exo-DNA-qPCR	54.6 (38.9–69.6)	100 (97.5–100.0)
			Total-DNA-qPCR	47.7 (32.5–63.3)	100 (97.5–100.0)
			Exo-DNA-ddPCR	61.4 (45.5–75.6)	100 (97.5–100.0)
			Total-DNA-ddPCR	75.0 (59.7–86.8)	100 (97.5–100.0)
Song et al. (35), China.	*N* = 42 (plasma) samples. TB 26 (PTB and EPTB), and no-TB 16. Gender M/F: 1/1.	Combination of several criteria (clinical diagnosis, ZNS, culture, and biopsy).	Rv3874(CFP10)-ddPCR	100	100
			Rv3874(CFP10)-qPCR	65 (44–83)	100
Aung et al. (36), Thailand.	*N* = 180 (sputum) samples. PTB: 74.	Combination of several criteria (clinical diagnosis, radiology, ZNS, GeneXpert MTB/RIF).	mpt64-ddPCR	100.0	95.3
			GeneXpert	82.4	100.0
Devonshire et al. (14), UK.	*N* = NR.	MTB plasmid containing rpoB and 16S rRNA genes and purified DNA from MTB laboratory reference (strain H37Rv).	ddPCR, qPCR, Xpert MTB/RIF	NR	NR

MTB, M. tuberculosis; PTB, pulmonary tuberculosis; EPTB, extrapulmonary tuberculosis; DTB, disseminated tuberculosis; MEC-TB, tuberculous meningitis; NTM, non-tuberculous mycobacteria; ZNS, Ziehl–Neelsen staining; H&E, hematoxylin and eosin; LJ, Lowenstein–Jensen; CSF, cerebrospinal fluid; interferon-γ release assay (IGRA); MGIT (Mycobacterium Growth Indicator Tubes); NAAT, nucleic acid amplification technologies; 95% CI, 95% confidence interval; FFPE, formalin-fixed and paraffin-embedded; CCDTGTB, Chinese Clinical Diagnosis and Treatment Guidelines, Tuberculosis; exoDNA, exosomal DNA; NR, not reported.

**Figure 2:**
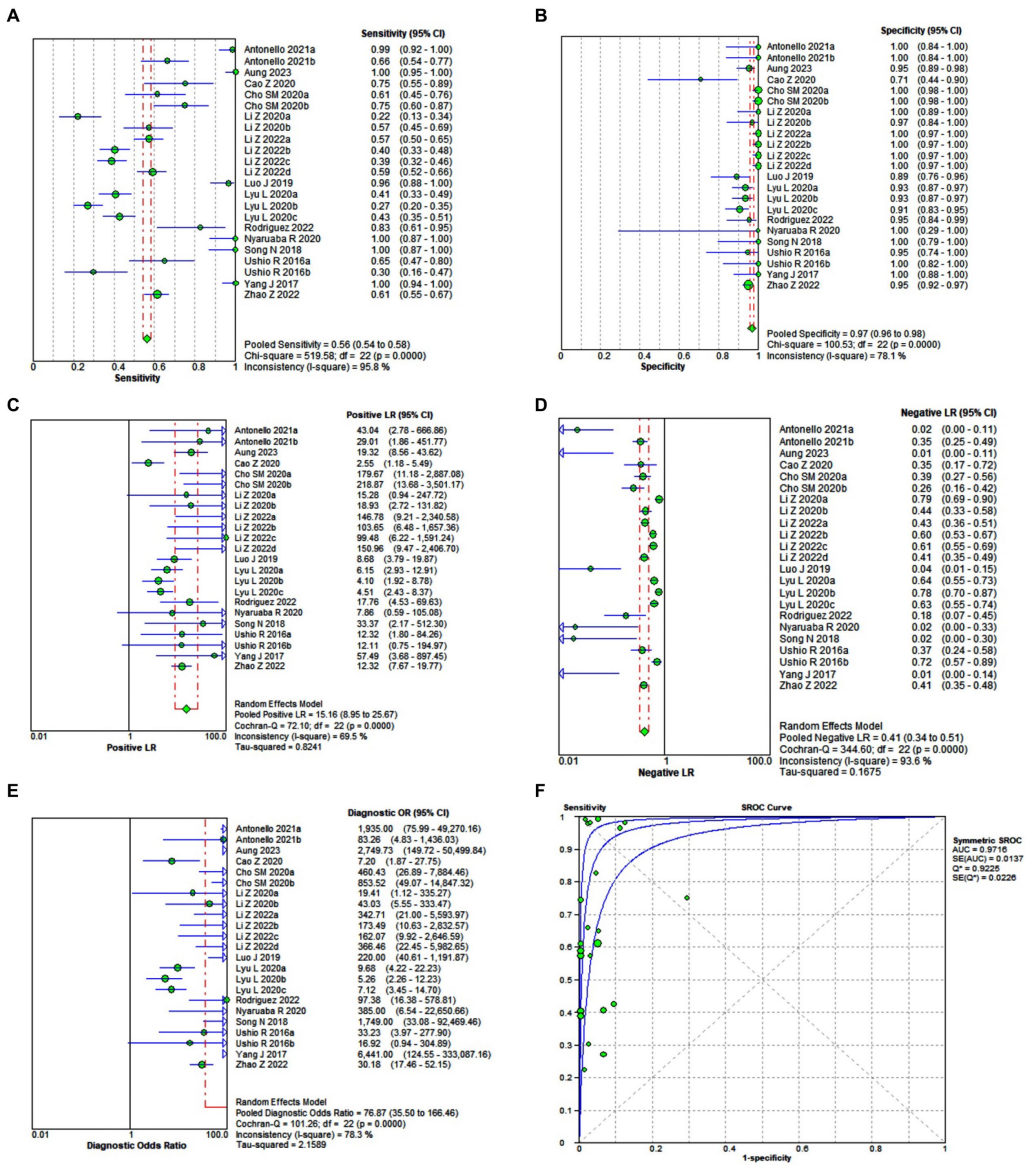
Forest plot of the pooled sensitivity **(A)**, specificity **(B)**, +LR **(C)**, −LR **(D)**, diagnostic OR **(E)**, and the area under the ROC **(F)** of droplet digital PCR (ddPCR) for diagnosing pulmonary or extrapulmonary tuberculosis.

Pooled diagnostic performance measures for droplet digital PCR (ddPCR). For ddPCR, sensitivity, specificity, +LR, −LR, and diagnostic OR were 0.562 (95% CI 0.541–0.583), 0.968 (95% CI 0.959–0.975), 15.155 (95% CI 8.947–25.670), 0.415 (95% CI 0.338–0.509), 76.869 (95% CI 35.497–166.46), respectively. The area under the ROC curve was 0.9716 (Figures 2A–F).

**Figure 3:**
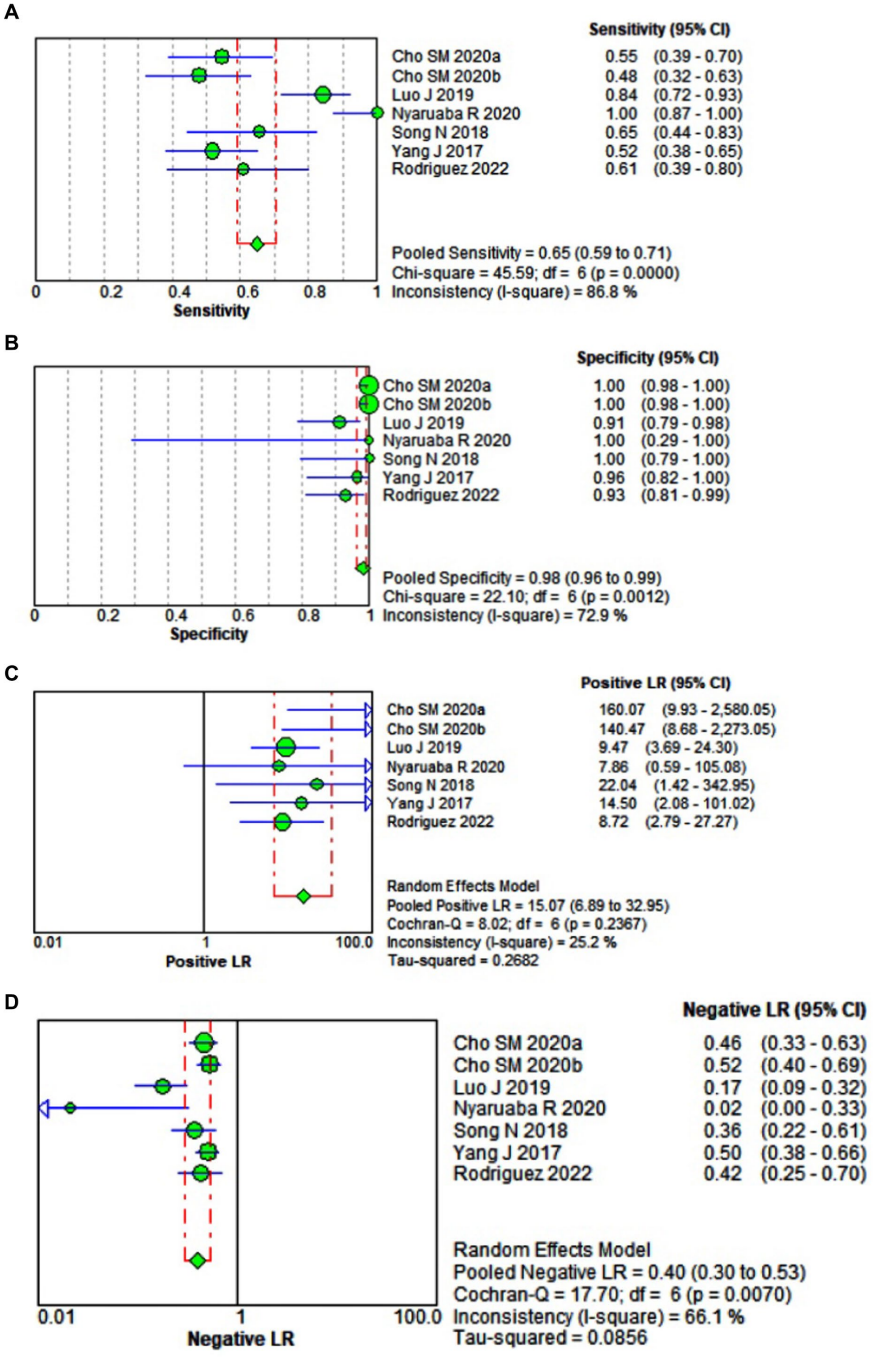

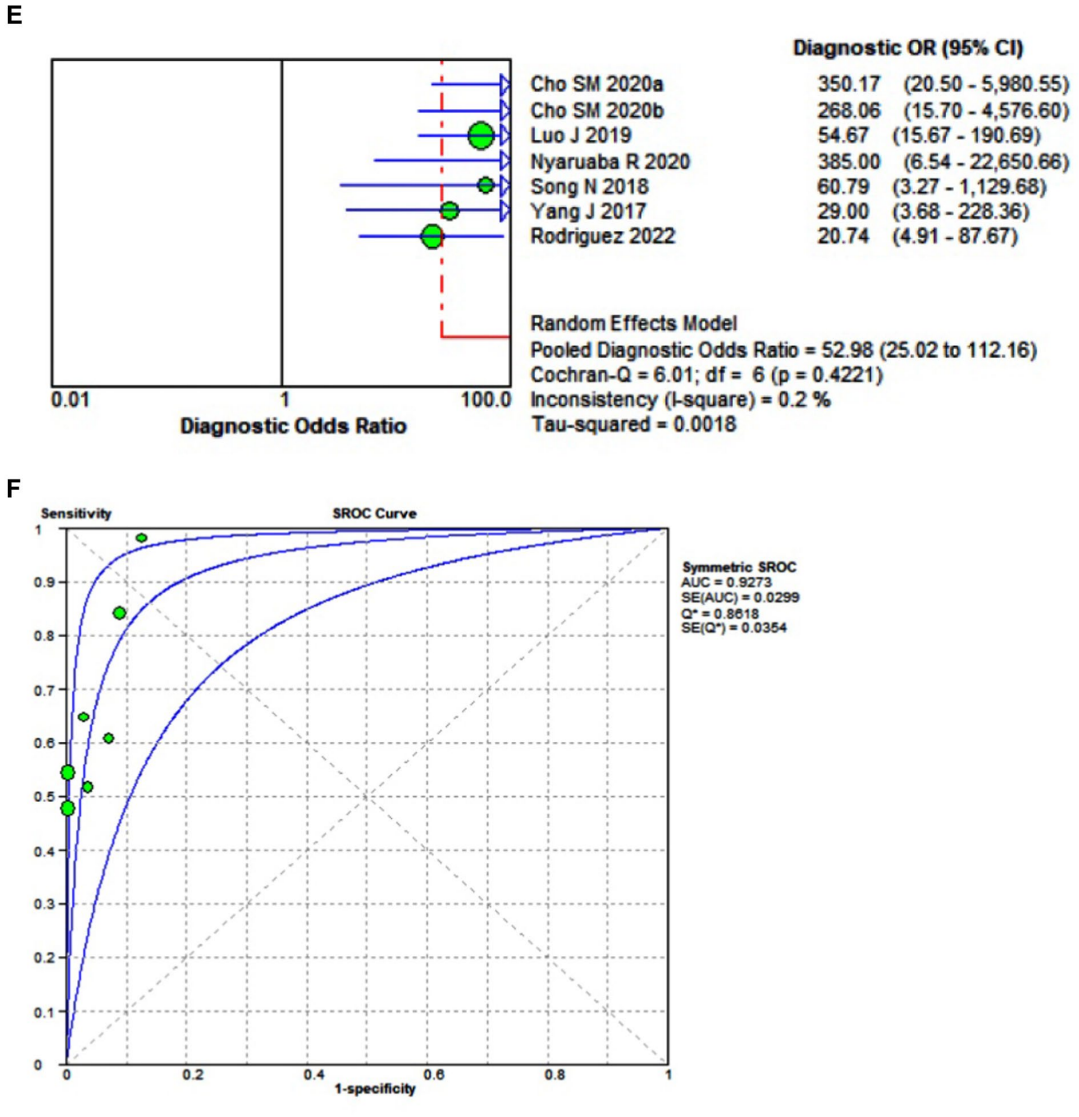
Forest plot of the pooled sensitivity **(A)**, specificity **(B)**, +LR **(C)**, −LR **(D)**, diagnostic OR **(E)**, and the area under the ROC **(F)** of quantitative Real Time-PCR (qPCR) for diagnosing pulmonary or extrapulmonary tuberculosis.

Pooled diagnostic performance measures for quantitative Real Time-PCR (qPCR). For qPCR, the sensitivity, specificity, +LR, −LR, and diagnostic OR were 0.650 (95% CI 0.590–0.706), 0.981 (95% CI 0.963–0.992), 15.067 (95% CI 6.889–32.952), 0.396 (95% CI 0.297–0.527), 52.975 (95% CI 25.021–112.16), respectively. The area under the ROC curve was 0.9276 (Figures 3A–F).

**Figure 4:**
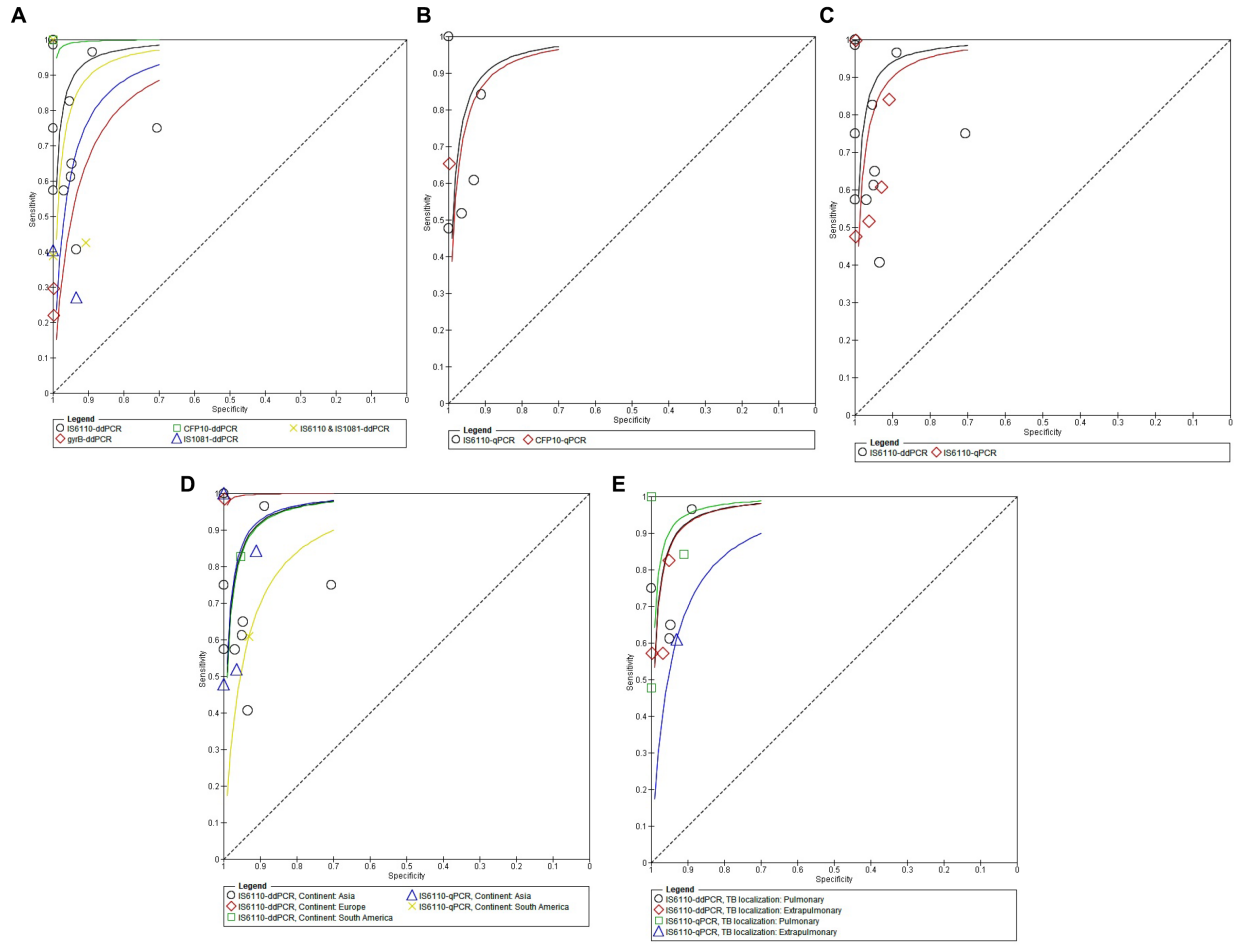
**(A)** The area under the ROC curve of ddPCR according to the type of insertion sequences for diagnosing pulmonary or extrapulmonary tuberculosis. **(B)** The area under the ROC curve of qPCR according to the type of insertion sequences for diagnosing pulmonary or extrapulmonary tuberculosis. **(C)** Areas under the ROC curve of ddPCR and qPCR according to the main type of insertion sequences employed for diagnosing pulmonary or extrapulmonary tuberculosis. **(D)** The areas under the ROC curve of ddPCR and qPCR according to the continent of origin of the study. **(E)** The areas under the ROC curve of ddPCR and qPCR according to the location of tuberculosis (PTB and EPTB).

Pooled diagnostic performance of ddPCR according to the type of insertion sequences. In the case of the CFP 10 sequence, the area under the ROC curve was 1.00, for the combined IS610 & IS1081 sequences the AUC was 0.9887, for the isolated IS6110 sequence the AUC was 0.9651, for the isolated IS108 sequence the AUC was 0.6915, and for the gyrB sequence it was 0.6214 (Figure 4).

Pooled diagnostic performance of quantitative Real Time-PCR (qPCR) according to the different types of insertion sequences. The AUC was higher for the IS6110 probe (AUC 0.9588) compared to the CFP10 sequence (AUC 0.9200) (Figure 4B).

Comparison of diagnostic performance between ddPCR and qPCR without including covariates. When we compared the AUCs and standard errors corresponding to ddPCR and qPCR using the Hanley & McNeil method, we found that there were statistically significant differences between them (AUC difference of 4.40%, *p* = 0.0020) (Figure 4C).

Comparison of diagnostic performance between ddPCR and qPCR with analysis of heterogeneity for the continent covariate of origin of the study. The AUCs were different for ddPCR and qPCR depending on the continent of origin of the study. ddPCR showed the highest AUC in a European study (AUC 0.971), while qPCR showed the highest AUC in Asia (AUC 0.9471). In general, both techniques showed lower AUC in the studies carried out in South America: ddPCR (0.969) and qPCR (AUC 0.875) (Figure 4D).

Comparison of diagnostic performance ddPCR and qPCR according to covariate location of tuberculosis. The AUCs were different for dPCR and qPCR depending on the location of tuberculosis. ddPCR and qPCR showed their highest AUC in patients with pulmonary tuberculosis studies. In patients with pulmonary tuberculosis, ddPCR showed a higher AUC than qPCR. However, for extrapulmonary tuberculosis with or without concomitant lung involvement, ddPCR had a higher AUC compared to qPCR (Figure 4E).

Most of the included studies had a low risk of bias according to the QUADAS-C tool (Table 2).

**Table 2:** QUADAS-C judgment of the included studies.

Study	Test	Risk of bias (QUADAS-C)			
		P	I	R	FT
Luo et al. (21)	IS6110-ddPCR	✓	✓	✓	✓
	IS6110-qPCR				
	Xpert-MTB/RIF				
Lyu et al. (22)	IS6110-ddPCR	✓	✓	✓	✓
	IS1081-ddPCR				
	IS6110 & IS1081-ddPCR				
Zhao et al. (23)	IS6110-ddPCR	✓	✓	✓	✓
Ushio et al. (24)	IS6110-ddPCR	✓	✓	✓	✓
	gyrB-ddPCR				
Cao et al. (25)	IS6110-ddPCR	✓	✓	✓	✓
Li et al. (26)	IS6110-ddPCR	✓	✓	✓	✓
	gyrB-ddPCR				
Yang et al. (28)	IS6110-ddPCR	✓	✓	✓	?
	IS6110-qPCR				
Li et al. (29)	IS6110-ddPCR	✓	✓	✓	✓
	IS1081-ddPCR				
	IS6110 & IS1081-ddPCR				
	IS6110 OR IS1081-ddPCR				
Antonello et al. (31)	IS6110-ddPCR	✓	✓	✓	✓
	rpoB-ddPCR				
	Xpert-MTB/RIF				
	ZNS				
Rodriguez and Villegas Chiroque (32)	ZNS	?	✓	✓	?
	IS6110-ddPCR				
	IS6110-qPCR				
Nyaruaba et al. (33)	IS6110 & IS1081-ddPCR	✓	✓	✓	✓
	IS6110-qPCR				
Cho et al. (34)	Exo-DNA-qPCR	✓	✓	✓	✓
	Total-DNA-qPCR				
	Exo-DNA-ddPCR				
	Total-DNA-ddPCR				
Song et al. (35)	Rv3874(CFP10)-ddPCR	✓	✓	✓	✓
	Rv3874(CFP10)-qPCR				
	ZNS				
	Sputum culture				
Aung et al. (36)	mpt64-ddPCR	✓	✓	✓	✓
	GeneXpert				
	ZNS				

## 4. Discussion

This study is the first systematic review and meta-analysis to analyze the performance of digital droplet PCR (ddPCR) compared to quantitative Real Time-PCR (qPCR) to diagnose pulmonary and extrapulmonary tuberculosis. According to our findings, ddPCR is a highly effective technique for diagnosing the disease in both forms. However, we believe that the most significant utility of this test lies in extrapulmonary disease, in which the other diagnostic methods show poor diagnostic performance. Diagnostic performance refers to the ability of a technique to detect and quantify disease in a biological sample.

Digital droplet PCR is a recently developed technology designed to rapidly detect and quantify minute amounts of genetic material in biological samples without using a standard curve. Furthermore, the evidence suggests it is relatively easy to implement and could significantly reduce diagnostic costs. Therefore, the extensive implementation of this test could be vital in diagnosing, treating, and controlling tuberculosis as a global public health problem. The latter is of particular interest in developing countries, which are the ones with the highest tuberculosis burden (8, 37, 38).

Comparative analysis of the diagnostic performance of ddPCR and qPCR. In general, regarding cumulative diagnostic performance measures, ddPCR, compared with quantitative Real Time-PCR (qPCR), had lower sensitivity, lower specificity, lower positive and negative likelihood ratio, and lower diagnostic odds ratio (OR). However, overall ddPCR, compared to qPCR, showed a higher area under the ROC curve (AUC). That is, ddPCR, compared to the qPCR, showed a greater capacity to discriminate between healthy and sick with tuberculosis (Figures 2A–F, 3A–F) since a test that has a higher AUC has a greater discriminant capacity between true positives and true negatives (39, 40). Similarly, when we compared both AUCs and their standard errors using the Hanley & McNeil method, we found that there are statistically significant differences between the AUCs of ddPCR and qPCR (AUC difference of 4.40%, *p* = 0.0020) (18) (Figure 4C).

Our results are concordant with other primary and secondary studies that have analyzed the diagnostic performance of ddPCR and qPCR in tuberculosis. Most of these studies highlight the advantages of ddPCR compared to qPCR for its ability to quantify target nucleic acid sequences in biological samples where genetic material is scarce (35, 37, 38).

Absolute quantification is a technique used to determine nucleic acids (expressed in copies per microliter) of various pathogens, including mycobacteria, in a given sample without needing a standard curve (41). Most studies on diagnostic tests for pulmonary and extrapulmonary tuberculosis using ddPCR employ this design. On the other hand, the different studies with ddPCR in tuberculosis have used different insertion sequences (IS). The most commonly used IS are those based on the IS6110, IS108, and gyrB genes (8, 14, 24, 37, 42). In the present review, in 12 of the 14 included studies, the authors used the sequence IS6110 in at least one of their tests.

In the present study, we found that, for ddPCR, the AUC was higher with the CFP 10 sequence (AUC 1.00), followed by the tests that used the sequences IS610 & IS1081 (AUC 0.99) combined, IS6110 (AUC 0.97) and IS108 (0.69) individually, and lower for the gyrB sequence (Figure 4). Whereas, for qPCR, the AUC was higher for the IS6110 probe (AUC 0.96), followed by the CFP10 insertion sequence (AUC 0.92) (Figure 4B). However, no study has previously compared the diagnostic performance of ddPCR and qPCR based on the different types of insertion sequences. Furthermore, it is essential to note that, according to our results and from a theoretical point of view, the ddPCR based on the CFP 10 insertion sequence would be a “perfect test” since its sensitivity, specificity, and AUC is 1 (43). However, this insertion sequence is not as common and of widespread use as the IS6110 sequence. In fact, it was only used in one study in our review (34). Therefore, this result should be interpreted with caution.

As previously mentioned, the most used insertion sequence for tuberculosis diagnosis is IS6110. However, when this sequence was used with conventional PCR, it presented some drawbacks. On the one hand, it has been shown that IS6110-based diagnosis is hampered by low copy numbers or repeated deletions of IS6110 (44). The use of ddPCR, given its high sensitivity, can be an excellent way to mitigate this problem (8). However, on the other hand, some clinical MTB isolates show IS6110 negativity, which can lead to false negative results. A feasible option would be to use the duplex ddPCR detection method developed and evaluated by Nyaruaba et al., combining the IS6100 and IS1081 insertion sequences for TB quantification. This detection method helps eliminate false negative results and dramatically reduces the detection cost, laying the foundation for the popularization of ddPCR (33).

Comparison of diagnostic performance between ddPCR and qPCR according to the continent of origin of the study. Comparative analysis of ROC AUCs between ddPCR and qPCR with heterogeneity analysis for the covariate continent of origin of the study showed that ddPCR had a maximum AUC in studies conducted in Europe (AUC 0.97). In contrast, qPCR showed a maximum AUC in studies conducted in Asia (AUC 0.95). In general, both techniques showed lower AUC in the studies carried out in South America: ddPCR (0.97) and qPCR (AUC 0.88) (Figure 4D). This lower diagnostic yield of molecular tests in low-resource countries is likely due to less training and experience with these techniques, which has also been shown to occur with other culture-based techniques and drug susceptibility testing (45).

Comparison of diagnostic performance between ddPCR and qPCR according to the location of tuberculosis. The AUCs were different for ddPCR and qPCR depending on the location of tuberculosis. ddPCR and qPCR showed their highest AUCs in patients with pulmonary tuberculosis studies. However, for extrapulmonary tuberculosis with or without concomitant lung involvement, ddPCR has a higher AUC compared to qPCR (Figure 4E). This is because pulmonary forms of tuberculosis usually have a higher load of mycobacteria than extrapulmonary forms, which allows their detection to be carried out with relative efficiency with both techniques. At the same time, ddPCR works better in extrapulmonary tuberculosis, in which samples are usually paucibacillary (46).

Various studies, including a study conducted in Peru in patients with clinical suspicion of extrapulmonary TB, showed that ddPCR has advantages over smear, culture, and qPCR for detecting low DNA copy numbers in samples from different origins of extrapulmonary tuberculosis patients. In biological samples from patients with extrapulmonary TB, ddPCR was 2.4 times more sensitive for detecting MTB than in smear microscopy and 7.3 times more sensitive compared with culture for detecting MTB. This was consistent with other studies (14, 25, 35). In this study, the authors reported that the mycobacterial load (DNA concentration in *n*g) was not directly related to the type of extrapulmonary sample (biopsy material, pleura or ascitic fluid, etc.) or the amount of DNA (copies/uL) extracted from the sample; since the samples with the highest concentration of nucleic acids did not always present the highest number of copies of mycobacteria. However, the authors reported that when they grouped and compared the type of tuberculosis and the DNA concentration, the results obtained from the samples presented a lower DNA concentration compared to that reported by Yang et al. (28).

Our systematic review and meta-analysis have some limitations: (1) the heterogeneity of the studies was significant, (2) the types of samples were very diverse, including from respiratory secretions, body fluids, plasma, etc., (3) we could not perform subgroup analyses according to other important variables such as the age, sex of the participants, and the type of tuberculosis (EPTB or PTB).

On the other hand, we highlight the following strengths of this work: (1) our search strategy was broad and complete, (2) it is the first meta-analysis that compares the diagnostic performance of ddPCR with qPCR, (3) we included only studies that adequately reported their results and that were compared against a reference test, (4) we only included primary studies that specifically evaluated the diagnosis of tuberculosis, (5) we performed sensitivity and heterogeneity analyses. Therefore, our results are more robust than any previously reported study.

Finally, we highlight that our findings have a potential application in the context of public health management and tuberculosis control as a global and priority health problem. The wide use of this test could allow an early diagnosis of the disease using samples obtained by non-invasive methods, such as plasma, urine, feces, etc. This would be particularly relevant in the extrapulmonary forms of tuberculosis, which are usually paucibacillary (10, 47, 48), since, according to our results, the extrapulmonary forms benefit the most from ddPCR. Although no studies have yet been published that have carried out economic evaluations (cost-effectiveness, cost-utility, cost-opportunity, etc.) of ddPCR in comparison with other diagnostic methods, we believe that our work could serve as a basis for carrying out this future research that in turn could help as a contribution for its implementation in our country in the future.

## 5. Conclusion

In conclusion, regarding the diagnosis of extrapulmonary tuberculosis, ddPCR, compared to qPCR, is a tool with a greater area under the ROC curve. Therefore, it shows a greater discriminant capacity to distinguish patients with and without extrapulmonary tuberculosis. Consequently, we recommend conducting primary studies using a larger sample size and various index diagnostic tests based on microbiology and molecular methods. Furthermore, these studies should preferably be carried out in countries with a higher burden of tuberculosis, especially extrapulmonary forms.

## Data availability statement

The original contributions presented in the study are included in the article/supplementary materials, further inquiries can be directed to the corresponding author.

## Author contributions

EM-R and MA-R: conceptualization and writing—original draft preparation. EM-R: methodology, software, visualization, supervision, project administration, and funding acquisition. EM-R, MA-R, and GV-T: validation and resources. EM-R and GV-T: formal analysis. MA-R and GV-T: investigation and writing—review and editing. All authors contributed to the article and approved the submitted version.

## Conflict of interest

The authors declare that the research was conducted in the absence of any commercial or financial relationships that could be construed as a potential conflict of interest.

## Publisher’s note

All claims expressed in this article are solely those of the authors and do not necessarily represent those of their affiliated organizations, or those of the publisher, the editors and the reviewers. Any product that may be evaluated in this article, or claim that may be made by its manufacturer, is not guaranteed or endorsed by the publisher.
