# Supplementary material for: Droplet digital PCR vs. quantitative real time-PCR for diagnosis of pulmonary and extrapulmonary tuberculosis: systematic review and meta-analysis
Source: Front Med (Lausanne). 2023 Aug 7;10:1248842. doi: 10.3389/fmed.2023.1248842 (PMC10440704; doi:10.3389/fmed.2023.1248842)
Supplement: Supplementary file 1 [file Table_1.DOCX]

**Table S1: Search strategy**

| **1. MEDLINE (PUBMED)** | | **Results** | **Date** |
| --- | --- | --- | --- |
| #1 | "Tuberculosis"[Mesh] | 203,938 | March 31/2023 |
| #2 | "Polymerase Chain Reaction"[Mesh] | 464,478 |  |
| #3 | "Digital” | 217,235 |  |
| #4 | #1 AND #2 AND #3 | 27 |  |
| **2. GOOGLE SCHOLAR** | | **Results** | **Date** |
| #1 | All in title "Tuberculosis" | 730,000 | March 31/2023 |
| #2 | All in title "Polymerase Chain Reaction" | 289,000 |  |
| #3 | All in title "Digital" | 3,610,00 |  |
| #4 | #1 AND #2 AND #3 | 325 |  |
| **3. SCOPUS** | | **Results** | **Date** |
| #1 | TITLE-ABS-KEY (“Tuberculosis”) | 350,255 | March 31/2023 |
| #2 | TITLE-ABS-KEY ("Polymerase Chain Reaction") | 1,191,912 |  |
| #3 | TITLE-ABS-KEY ( "digital" ) | 1,420,364 |  |
| #4 | #1 AND #2 AND #3 | 104 |  |
| **4. SCIENCEDIRECT** | | **Results** | **Date** |
| #1 | "Tuberculosis" | 160,827 | March 31/2023 |
| #2 | "Polymerase Chain Reaction" | 14,086 |  |
| #3 | "digital" | 16,681 |  |
| #4 | #1 AND #2 AND #3 | 3 |  |
| **5. EMBASE** | | **Results** | **Date** |
| #1 | 'tuberculosis'/exp | 321,535 | March 31/2023 |
| #2 | 'polymerase chain reaction'/exp | 1,147,430 |  |
| #3 | 'digital' | 137,732 |  |
| #4 | #1 AND #2 AND #3 | 96 |  |
| **6. WEB OF SCIENCE** | | **Results** | **Date** |
| #1 | ALL=("tuberculosis") | 162,863 | March 31/2023 |
| #2 | ALL=(“Polymerase Chain Reaction”) | 322,996 |  |
| #3 | ALL=("digital") | 583,278 |  |
| #4 | #1 AND #2 AND #3 | 23 |  |
| **7. COCHRANE LIBRARY** | | **Results** | **Date** |
| #1 | Title Abstract Keyword "tuberculosis" | 7,435 | March 31/2023 |
| #2 | Title Abstract Keyword "Polymerase Chain Reaction" | 10,339 |  |
| #3 | Title Abstract Keyword "digital" | 22,268 |  |
| #4 | #1 AND #2 AND #3 | 0 |  |

**Table S2: Excluded studies**

| Excluded study | Reason for exclusion |
| --- | --- |
| Zheng Y, Xia H, Bao X, Zhao B, He P, Zhao Y. Highly Sensitive Detection of Isoniazid Heteroresistance in Mycobacterium Tuberculosis by Droplet Digital PCR. Infect Drug Resist. 2022;15:6245-6254. doi: 10.2147/IDR.S381097. | It does not assess the outcome of interest. |
| Whale AS, Nixon G, Wilson P, Jones G, McHugh TD, Foy CA, Huggett JF. Highly reproducible absolute quantification of Mycobacterium tuberculosis complex by digital PCR. Anal Chem. 2015 Apr 7;87(7):3706-13. doi: 10.1021/ac5041617. | It does not assess the outcome of interest. |
| Yamamoto M, Ushio R, Watanabe H, Tachibana T, Tanaka M, Yokose T, et al. Detection of Mycobacterium tuberculosis-derived DNA in circulating cell-free DNA from a patient with disseminated infection using digital PCR. Int J Infect Dis. 2018;66:80-2. DOI: https://doi.org/10.1016/j.ijid.2017.11.018 | Case report. |
